# Radiographic parameter(s) influencing functional outcomes following angular stable plate fixation of proximal humeral fractures

**DOI:** 10.1007/s00264-021-04945-2

**Published:** 2021-03-23

**Authors:** Marc-Daniel Ahrend, Luise Kühle, Stephan Riedmann, Sonja D. Bahrs, Christian Bahrs, Patrick Ziegler

**Affiliations:** 1 Department of Trauma and Reconstructive Surgery, BG Unfallklinik Tuebingen, Eberhard Karls University Tuebingen, Schnarrenbergstrasse 95, 72076 Tuebingen, Germany; 2 AO Research Institute Davos, Davos, Switzerland; 3 Department of Orthopedics and Trauma Surgery, St Josefs Hospital, Wiesbaden, Germany; 4 Diakonie Klinikum Stuttgart, Orthopädische Klinik Paulinenhilfe, Stuttgart, Germany; 5 Department of Diagnostic and Interventional Radiology, University Hospital Tuebingen, Tuebingen, Germany; 6 Department of Orthopedic Surgery and Traumatology, Schön Klinik Neustadt in Holstein, Neustadt in Holstein, Germany

**Keywords:** Angular stable plate fixation, Proximal humeral fracture, Functional outcome, Radiographic parameters

## Abstract

**Purpose:**

Radiographic parameters which correlate with poor clinical outcome after proximal humeral fractures could be helpful indicators to answer the question which patients should be followed up closer. Moreover, during surgery, radiographic parameters correlating with unfavourable outcome should be avoided. The primary aim of the study was to compare radiographic measurements between the injured and the contralateral, uninjured shoulder. The secondary aim was to correlate these radiographic parameters with post-operative shoulder function.

**Methods:**

Fifty-eight patients (age: 55.6 ± 14.4 years, age at surgery) following angular stable plate fixation of a proximal humeral fracture (2-part fractures according to Neer: 24, 3-part: 25, 4-part: 9) were included in this retrospective cohort study. All patients were followed up at least six years (7.9 ± 1.4 after surgical intervention). During follow-up examination, the Constant score (CS) was assessed, and radiographs of both shoulders were taken. Radiographs were analyzed regarding lateral humeral offset, distance between tuberculum and head apex, head diameter, head height, perpendicular height, perpendicular center, vertical height, and angles between head and humeral shaft (CCD and HSA). These parameters were compared between the injured and uninjured shoulder. The cohort was divided in two groups: patients with a CS category of excellent/good and satisfying/worse. Both groups were tested regarding differences of demographic and radiographic parameters.

**Results:**

The distance between tuberculum and head apex (2.6 ± 3.4 mm vs. 4.3 ± 2.1 mm; *p* = 0.0017), the CCD (123.1 ± 12.9° vs. 130.1 ± 7.3°; *p* = 0.0005), and the HSA (33.1 ± 12.8° vs. 40.1 ± 7.3°; *p* = 0.0066) were significantly smaller on the treated shoulder compared to the uninjured side. Patients reached a Constant score of 80.2 ± 17.4 (95% CI 75.6–84.8) points. Regarding outcome categories of the Constant score, 46 patients had a good to excellent outcome, and 12 patients had a satisfying or bad outcome. The comparison of these groups revealed that patients with inferior outcome in the long-term follow-up were older, female, had a more complex fracture type (AO classification), smaller lateral humeral offset, smaller head diameter and height, lower perpendicular height, and lower CCD and HSA angles.

**Conclusion:**

If the abovementioned parameters cannot be restored sufficiently during surgery, (reversed) shoulder arthroplasty might be a better solution to reach good post-operative outcome. Moreover, patients presenting these radiographic characteristics in the follow-up, older patients, and patients with a more complex fracture type should be followed up closer to possibly prevent poor shoulder function.

Trial registration: 83 250/2011BO2

## Introduction

Proximal humeral fractures are common fractures with an increasing incidence [1–5]. Decision between operative and conservative treatment depends on various factors such as patient age, pre-existing conditions, degree of dislocation, fracture morphology, and patient’s expectation. However, no consensus about the gold-standard treatment of proximal humeral fractures is present [6]. Younger or active patients with good bone quality with a multifragmentary fracture and severe fracture dislocation are commonly treated with open reduction and internal fixation [7, 8]. The majority of patients reaches good subjective and objective results following angular stable plates [9]. However, a systematic review of 514 patients with proximal humeral fractures treated with locking plates found a complication rate of 48.8% [9]. Previous studies found that initial poor anatomic reduction, especially of the medial column, can enhance failure risk following plate fixation [10, 11]. Several radiographic parameters might influence outcome measures. However, no consensus about radiographic parameters especially in comparison to the contralateral uninjured side is present. As patients with humeral fractures are often elderly, follow-up examinations are often difficult to organize [12]. Radiographic parameters which correlate with poor clinical outcomes could be helpful indicators to answer the question which patients should be followed up closer. Moreover, during surgery, these radiographic parameters which correlate with unfavourable outcome should be restored anatomically.

Therefore, the primary aim of the present retrospective study was to compare radiographic measurements (lateral humeral offset, distance between tuberculum and head apex, head diameter, head height, perpendicular height, perpendicular center, vertical height, and inclination angles (CCD, HSA)) between the injured and the contralateral, uninjured shoulder. The secondary aim was to correlate these radiographic parameters with shoulder function. We hypothesized that radiographic parameters differ significantly between both shoulders and that these parameters differ significantly between patients with good and poor shoulder function.

## Materials and methods

### Study design and patient recruitment

The present cohort study (level IV) was conducted in a level-1 trauma centre and approved by the local ethics committee. Within a four year timeframe, 191 patients with proximal humeral fractures were treated with angular stable plate fixation.

Exclusion criteria were as follows: patient’s death, no accessibility, paresis of the upper extremity, severe dementia, injury of the contralateral shoulder, injury during the post-operative interval due to an additional trauma, or change of therapeutic concept of an anatomical reconstruction of the humeral head during the follow-up period (e.g., revision surgery with arthroplasty). Only patients with a follow-up of at least six years were included. Moreover, only patients were included if radiographs were assessed from both shoulders at the time of follow-up.

**Fig. 1:**
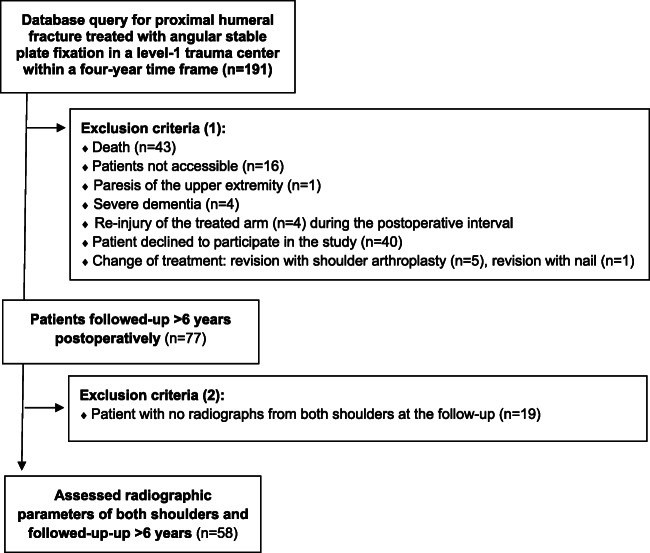
Patient flow chart

The patient flow chart is presented in Fig. 1.

### Participants

The final data set comprised 58 patients (male: 26, female: 32) with a mean age of 55.6 ± 14.4 years (range: 17.7 to 101.3 years, age at surgery). All patients were followed up at least six years (7.9 ± 1.4 (range: 6.2 to 11.1 years) after surgical intervention. According to the Neer classification [13], the cohort consisted of 24 2-part fractures, 25 3-part fractures, and 9 4-part fractures. According to the AO/OTA classification [14], these fractures were classified as 24 A, 22 B, and 12 C fractures. All fractures were treated with an angular stable plate (PHILOS® plate, DePuy Synthes, West Chester, Pennsylvania, USA or Non-Contact Bridging plate, Zimmer, Germany GmbH, Freiburg, Deutschland, or 4.5-mm T-plate (Stratec Medical, Oberdorf, Schweiz).

### Surgical treatment and postoperative care

The surgery was performed by using either a deltoid split, a deltoideo-pectoral, or an anterolateral approach according to Bigliani. Extensive fracture exposure was avoided trying to perform the surgery as less invasive as possible [15]. Surgical procedure is described by Bahrs et al. [16] as well as Ziegler et al. [12]. Fracture reduction was achieved by different techniques [17]: Nonabsorbable sutures at the humeral insertion of the rotator cuff were used for indirect reduction. Moreover, K-wires were used to maneuver fragments as joysticks and temporarily fixate the reduction. Head fragments with impaction were reduced by an elevator or chisel underneath the humeral head.

Following surgery, the shoulder was immobilized using a shoulder sling during the first seven days. Afterwards for four weeks, active-assisted movement including up to 90° of abduction and flexion was allowed. All patients received physiotherapeutic treatment after the operation.

### Outcome variables

**Fig. 2:**
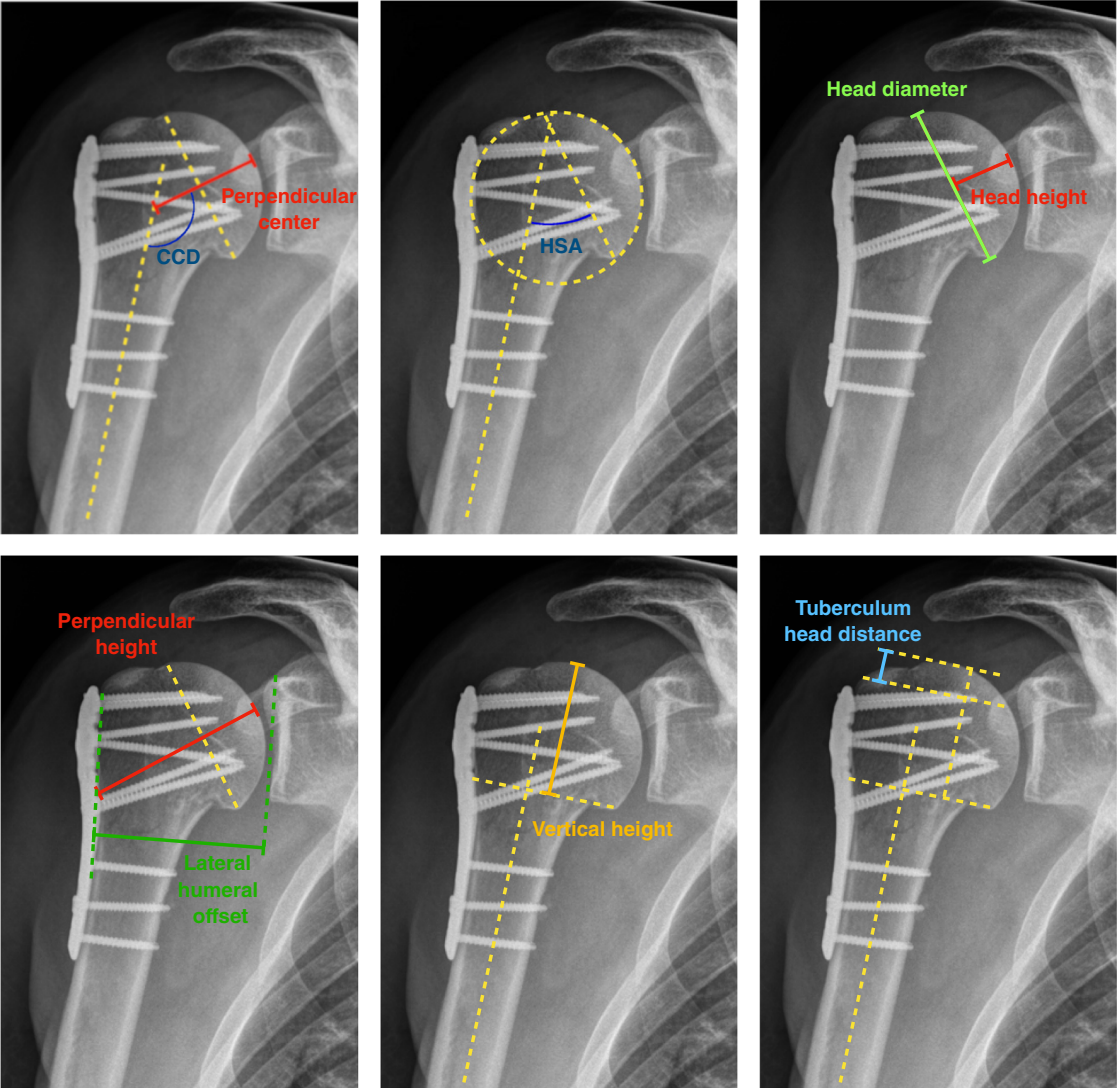
Overview of the measured radiographic parameters (dashed lines help to determine each parameter): Lateral humeral offset, distance between tuberculum and head apex, head diameter, head height, perpendicular height, perpendicular center, vertical height, and angles between head and humeral shaft (CCD and HSA)

Antero-posterior and lateral scapula views were assessed of both shoulders under supervision of a specialist in orthopaedic surgery and standardized position of the patient at least six years after surgery. Measurements were performed with Impax Viewer 6, AgfaHealthcare©, Mortsel Belgium. Plate and screw sizes were used as scales to avoid magnification failure. Radiographs were analyzed by one consultant who was well experienced in trauma care. The following parameters were assessed (see Fig. 2). Dashed lines helped to determine each parameter: perpendicular height (distance from the head apex to the lateral shaft cortex, distance is perpendicular to the head diameter), lateral humeral offset (distance between two parallel lines: first line runs from the tip to the bottom of the glenoid, and the second line is parallel to the first line and is tangent to the humeral lateral border midpoint), vertical height (distance from the most proximal to the most distal point of the articular surface of the head, distance is parallel to the humeral shaft axis), tuberculum head distance (distance between the level of the tuberculum and the level of the head apex; both levels are perpendicular to the humeral shaft), head diameter (distance between the inferior and superior cortex of the articular surface), head height (distance of the middle of the head diameter to the articular cortex, distance is perpendicular to the head diameter), perpendicular center (distance of the middle of the head diameter to the shaft axis, the distance is perpendicular to the head diameter), CCD (angle between perpendicular centre and shaft), and HSA (angle between shaft and head diameter).

The Constant score assesses pain and shoulder function during daily activities, range of motion, and shoulder strength [18]. The Constant score was categorized as excellent from 100 to 86 points, good from 71 to 85 points, satisfying from 70 to 56 points, and worse lower than 56 points [19]. Complications were documented retrospectively at the follow-up time point. Humeral head necrosis, surgical site infection, screw perforation, and shoulder impingement were documented.

### Statistical analysis

The continuous scores and radiographic parameters were described descriptively as mean ± standard deviation (95% confidence interval). Categories of scores and complications (occurrence of screw perforation, humeral head necrosis, infection, and shoulder impingement) were described as *n* (%). Shapiro-Wilk test was used to check if data was normally distributed. Radiographic parameters were compared between injured and uninjured shoulder. The cohort was divided in two groups: patients with a CS category of excellent and good as well as satisfying and worse. Both groups were tested regarding anthropometric- and fracture-related differences as well as abovementioned radiographic parameters. The *t* test was used in case of normally distributed data. If data were not normally distributed, we used the Mann-Whitney *U* test. Chi-square test was used to test the differences between categorical variables.

## Results

Nineteen (32.8%) patients underwent implant removal after osseous consolidation. 34.5% (*n* = 20) of patients had either one or more of the following complications. The authors found one surgical site infection (1.7%). Four patients (6.9%) showed necrosis of the humeral head. Six patients (10.3%) had screw perforations (2 caused by necrosis of the femoral head), and 12 patients (21.0%) had joint stiffness or impingement following surgery.

Analysis of radiological parameters between the injured and uninjured shoulder revealed that the distance between tuberculum and head apex (2.6 ± 3.4 mm vs. 4.3 ± 2.1 mm; *p* = 0.0017) as well as the CCD-angle (123.1 ± 12.9° vs. 130.1 ± 7.3°; *p* = 0.0005) and the HSA (33.1 ± 12.8° vs. 40.1 ± 7.3°; *p* = 0.0066) were significantly smaller compared to the uninjured side after surgery (see Table 1).

**Table 1:** Differences of radiographic parameters between uninjured and injured shoulder (mean ± standard deviation (95% CI))

	Injured shoulder	Uninjured shoulder	*p* value
Lateral offset (mm)	48.4 ± 4.9 (47.1–49.7)	48.3 ± 5.6 (46.8–49.8)	0.8641
Distance tuberculum—head (mm)	2.6 ± 3.4 (1.7–3.5)	4.3 ± 2.1 (3.7–4.8)	0.0102*
Head diameter (mm)	47.9 ± 4.6 (46.7–49.1)	47.6 ± 5.6 (46.1–49.1)	0.9121
Head height (mm)	20.4 ± 2.6 (19.7–21.1)	21.3 ± 3.8 (20.2–22.3)	0.2213
Vertical height (mm)	43.5 ± 4.8 (42.2–44.7)	42.0 ± 5.0 (40.7–43.3)	0.1303
Perpendicular height (mm)	50.8 ± 6.3 (49.1–52.5)	50.2 ± 6.5 (48.5–52.0)	0.5903
Perpendicular center (mm)	32.4 ± 5.5 (30.9–33.8)	32.6 ± 14.0 (28.9–36.3)	0.1599
CCD-angle	123.1 ± 12.8 (119.7–126.5)	128.5 ± 13.3 (125.0–132.1)	0.0133*
HSA-angle	33.1 ± 12.8 (29.7–36.5)	40.1 ± 7.3 (38.1–42.0)	0.0066*

Patients reached a Constant score of 80.2 ± 17.4 (95%CI 75.6–84.8) of 100 possible points in the follow-up examination. Regarding outcome categories of the Constant score, 46 patients out of 58 patients had a good to excellent outcome. Twelve patients showed a satisfying or poor outcome.

The comparison of these groups showed that patients with excellent and good Constant scores are younger. Moreover, female patients and patients with a more complex fracture type according to the AO classification have an inferior clinical outcome in the long-term follow-up. Regarding radiographic parameters, patients with an excellent and good score have significantly higher lateral humeral offset, a larger head diameter, and head height. Moreover, the perpendicular height, the CCD, and the HSA angle are significantly higher (Table 2).

**Table 2:** Differences of anthropometric fracture-related and radiographic parameters between patients with excellent/good and satisfying/worse shoulder function according to the Constant score (mean ± standard deviation (95%CI))

	Patients with excellent and good Constant score	Patients with satisfying and worse Constant score	*p* value
Anthropometric and fracture variables			
Age at follow-up	60.4 ± 13.1 (56.5–64.3)	68.3 ± 9.8 (62.1–74.6)	0.0304*
Gender	Female: 68.8% Male: 92.3%	Female: 31.3% Male: 7.7%	0.0276*
Fracture type according to Neer	2-part: 91.7% (*n* = 22) 3-part: 76.0% (*n* = 19) 4-part: 55.6% (*n* = 5)	2-part: 8.3% (*n* = 2) 3-part: 24.0% (*n* = 6) 4-part: 44.4% (*n* = 4)	0.0641
Fracture type according to AO	A: 91.7% (*n* = 22) B: 81.8% (*n* = 18) C: 50.0% (*n* = 6)	A: 8.3% (*n* = 2) B: 18.2% (*n* = 4) C: 50.0% (*n* = 6)	0.0136*
Radiographic measurement			
Lateral humeral offset	49.20 ± 5.00 (47.72–50.69)	45.36 ± 3.35 (43.23–47.49)	0.0041*
Distance tuberculum—head (mm)	3.15 ± 2.85 (2.30–3.99)	0.33 ± 1.30 (−2.53–3.19)	0.0613
Head diameter	48.56 ± 4.69 (47.17–49.95)	45.42 ± 3.87 (42.96–47.88)	0.0268*
Head height	20.81 ± 2.56 (20.05–21.57)	18.67 ± 2.11 (17.33–20.01)	0.0071*
Vertical height	43.01 ± 4.87 (41.57–44.45)	45.17 ± 4.48 (42.32–48.02)	0.1612
Perpendicular height	51.99 ± 6.21 (50.15–53.84)	46.24 ± 4.92 (43.12–49.37)	0.0027*
Perpendicular center	33.03 ± 5.00 (31.54–34.51)	29.84 ± 6.79 (25.53–34.15)	0.1495
CCD	125.40 ± 11.97 (121.85–128.96)	114.10 ± 13.09 (105.78–122.42)	0.0093*
HSA	35.35 ± 11.98 (31.79–38.91)	24.26 ± 13.23 (15.85–32.67)	0.0101*

## Discussion

The primary aim of the present study was to describe difference of radiographic parameters compared to the uninjured shoulder. As hypothesized, following surgery, the shape and size of the treated proximal humeral fracture differed significantly regarding distance between tuberculum and head as well as angles between the head and the humerus shaft (CCD and HSA). Correlating radiographic parameters with clinical outcomes scores, patients with excellent and good outcomes have a higher lateral humeral offset, a larger head diameter, a larger head height, and a larger perpendicular height. Moreover, CCD and the HSA angle were larger in patients with superior outcomes.

Inferior clinical outcomes and surgical complications following osteosynthesis of proximal humeral fractures can be dependent on patient-specific, fracture-related, and treatment-related factors. Boesmueller et al. [20] analyzed risk factors for humeral head necrosis and non-union after plating in proximal humeral fractures of 154 patients. They found 16.2% of patients with necrosis, 13% of non-union, and screw cut out in 27.9% of cases. They found statistically significant correlations between head necrosis and the severity of fracture, non-union and smoking, as well as screw-cut out and higher age. This is in accordance to other studies which described osteoporosis, medial side comminution, and initial varus displacement as predictor of failure, loss of reduction, and lower functional outcomes [11, 21, 22].

Hardemann et al. [23] found a failure rate of 15.3% in 307 angular stable osteosynthesis with nail or plate of the proximal humerus with a mean follow-up of 4.3 years. Hardemann et al. [23] compared the post-operative functional outcome with patient characteristics, fracture characteristics, and quality of reduction. Older patients, more displaced fractures, AO type C fractures, varus fracture configuration, and reduced head vascularity led to worse outcomes. The assessment of quality of reduction revealed that a fracture gap of more than 2 mm on the medial side shows higher failure rates (27.8% vs. 6.8% gap less than 2 mm) which were defined as head necrosis, loss of reduction, screw cut out, deep infection, and malunion. Moreover, a humeral head varus malalignment of > 10° increased failure rates (26.4% vs. 8.7% in the neutral reduction) and decreased functional outcome scores (70.8 vs. 77.7 points in the American Shoulder and Elbow Surgeon Score (ASES)). A cumulative displacement of more than 10 mm showed high failure rates of 31.0% and ASES scores of 70.5 points compared to less than 10-mm displacement with a failure rate of 4.5% and ASES scores of 78.8 points.

Schnetzke et al. [24] analyzed parameters of fracture reduction and its influence on outcomes after locked plate fixation of proximal humeral type-C fractures. Ninety-eight patients with a mean follow-up of 3.1 ± 1.5 years were included. Cranialization of the greater tuberosity of > 5 mm, head shaft displacement of > 5 mm, and valgus head-shaft alignment (> 150°) increased the risk for a Constant score less than 50% by twofold to threefold.

In our study, we analyzed different radiographic parameters at the long-term follow-up as most of the abovementioned studies present short- to mid-term follow-ups. Parameters were selected based on parameters used in shoulder arthroplasty [25]. Descriptions and names of radiographic parameters are heterogenous, and interindividual variability is described for several bones [26–28]. Kamer et al. [28] described large variations of size, shape, and bone stock distribution in the proximal humeri of 58 specimens. Shape variation was primarily the result of variation of the humeral head inclination and the shaft portion. Robertson et al. [29] analyzed CT scans of sixty cadaveric humeri and measured anatomical parameters. They found comparable values compared to the healthy side in our study, for example, for head height 19 ± 2 (range: range: 15–24 mm), humeral inclination (HSA) 41 ± 3 (range: 34–47° mm), and distance between tuberculum and head apex 6 ± 2 (range: 3–8 mm).

A risk factor analysis could not be calculated as radiographic parameters were not assessed directly following surgery. However, our results point out radiographic parameters which might lead to poor radiographic and functional outcome. The distance between tuberculum and humeral head, CCD-angle, and the HSA were significantly different between both shoulders. Regardless of the uninjured shoulder, we found that patients with an excellent and good Constant score have significant higher lateral humeral offset, a larger head diameter, and head height as well as higher perpendicular height, larger CCD, and HSA angles. These parameters should be carefully checked during surgery and if necessary corrected as they can lead to superior or inferior functional outcomes in the long term. Our results suggest that surgeons should aim to achieve a fracture reduction with a more valgus shaft to head relation (measured with CCD and HSA), a higher lateral humeral offset which results in less needed abduction forces to elevate the arm, and a larger head configuration (measured with head height, head diameter, or perpendicular height).

Best possible fracture reduction can be achieved by different techniques. The applied technique is dependent on bone quality, fracture pattern, and surgeon’s preferences. In osteoporotic bones and more fragmentary fracture patterns, we recommend inserting nonabsorbable sutures at the insertion of rotator cuff tendons. The sutures can be used to manipulate, reduce, and to put a potential tuberosity fragment in continuity with the shaft fragment. The reduction can be supported by the use of a periosteal elevator in combination with ligamentotaxis, which can be achieved by manual axial traction of the humeral shaft. Using axial traction is especially advisable to restore the medial cortices as direct visualization is not possible. The use of an elevator is beneficial in fractures with an impacted head fragment, too. Temporarily fixating k-wires inserted through the fracture components represent another reduction technique. These k-wires can also be used as joysticks. Reduction of the commonly medial displaced humeral shaft in subcapital fractures can be achieved by applying longitudinal traction and lateral pull in combination with positioning the plate laterally on the humeral. By this means, the shaft is pulled laterally by introducing a screw distal to the fracture line [17, 30].

Standardized anterior-posterior radiographs are necessary to verify sufficient reduction during surgery. With the help of new technology, intra-operative imaging with the c-arm enables the surgeon to measure these radiological parameters intra-operatively. As interindividual differences of the humeral anatomy are present, radiographs of the uninjured contralateral side could also be beneficial especially in complex fractures. Moreover, our results showed that more complex fractures in accordance to the AO, female, and older patients are more likely to have inferior shoulder function in the long term. Therefore, patients with abovementioned radiographic, anthropometric, and fracture-related parameters should be followed up more carefully. Due to the limited sample size, multivariate analysis of radiographic, anthropometric, and fracture-related parameters was not valid. Moreover, sub-analysis of the influence of the analyzed radiographic parameters on the functional outcomes of the different fracture types could not be calculated and is of interest for further studies. Determined radiographic parameters relate on each other, and also age-related changes could have influence abovementioned findings. Changes of radiographic parameters due to loss of reduction can occur in the first 3 months. For example, changes of the angle between shaft and head are in average 4.9° ± 3.3° between one and three months post-operatively. At a later stage, these changes are smaller. Between six and 12 months post-operatively, the angle between shaft and head angle changes just by 0.3 ± 0.5° [31].

In our study, patients reached a Constant score of 80.2 ± 17.4 (95% CI 75.6–84.8) of 100 possible points. These results are comparable to previous published studies [32–35]. The meta-analysis by Dai et al. [34] reported Constant scores between 65.2 and 83.9 points and between six to 60 months following locking compression plate fixation of proximal humeral fractures [32, 33]. Ockert et al. [35] analyzed 43 fractured shoulders with a median follow-up of ten years. In average, patients reached a mean constant score of 75.3 points.

Limitations of the present study are the missing power analysis and a high rate of excluded patients. The low follow-up rate can be explained with the initial old patient age. Forty-three patients died until follow-up examination, and 40 patients declined to participate, mainly because of limited mobility. However, the present study could include 58 patients as outlined in Fig. 1. The strength of the study is the combined assessment of clinical long-term outcomes at least six years post-operatively and the assessment of radiographic parameters in comparison to the contralateral side. However, radiographic parameters were only measured on radiographs. CT imaging would have given three-dimensional information of radiographic parameters and is more reliable to assess prognostic factors of reduction in proximal humerus fractures [36]. Interrater reliability was not measured, but the observer was blinded regarding shoulder function.

## Conclusion

Six years after surgical intervention with an angle stable plate of proximal humeral fractures, we found differences between the shape and the size of the treated shoulder in comparison to the contralateral, uninjured shoulder. The distance between the tuberculum and the head as well as angles between the head and the humerus shaft (CCD and HSA) differed significantly. We found that patients with inferior shoulder function have smaller lateral humeral offset, head diameter, head height, and perpendicular height as well as smaller angles between shaft and head. These parameters should be restored anatomically during surgery. If a sufficient reduction cannot be achieved, (reversed) shoulder arthroplasty might be the better solution. Moreover, patients presenting the abovementioned radiographic characteristics in the follow-up radiographs, patients with poor bone quality, and patients with a more complex fracture-type should be followed up closer to possibly prevent poor shoulder function.
